# A PET microdosing study with the P-glycoprotein inhibitor tariquidar

**DOI:** 10.1186/2050-6511-13-S1-A17

**Published:** 2012-09-17

**Authors:** Martin Bauer, Markus Zeitlinger, Cécile Philippe, Johann Stanek, Wolfgang Wadsak, Markus Mitterhauser, Georgios Karanikas, Markus Müller, Oliver Langer

**Affiliations:** 1 Department of Clinical Pharmacology, Medical University of Vienna, 1090 Vienna, Austria; 2 Department of Nuclear Medicine, Medical University of Vienna, 1090 Vienna, Austria; 3 Health and Environment Department, AIT Austrian Institute of Technology GmbH, 2444 Seibersdorf, Austria

## Background

The adenosine triphosphate-binding cassette transporters P-glycoprotein (Pgp) and breast cancer resistance protein (BCRP) restrict absorption and body distribution and promote excretion of several clinically used drugs. Tariquidar (XR9576) is a potent third-generation dual Pgp and BCRP inhibitor, which is currently tested in clinical trials to overcome chemoresistance of tumors and to enhance brain distribution of Pgp/BCRP substrate drugs. We performed a positron emission tomography (PET) microdosing study with carbon-11-labelled tariquidar ([^11^C]tariquidar) which aimed at assessing the brain distribution of [^11^C]tariquidar in healthy volunteers.

## Methods

Six healthy subjects received an i.v. bolus injection of approximately 400 MBq of [^11^C]tariquidar containing less than 30 µg of unlabelled tariquidar. Then, dynamic brain PET scans and arterial blood sampling were performed. Radiolabelled metabolites of [^11^C]tariquidar in plasma were measured with a solid-phase extraction/HPLC assay. Brain activity uptake was expressed as the ratio of the area under the whole brain grey matter time-activity curve to the area under the plasma time-activity curve from time 0 to 60 min (AUC_0–60 brain_/AUC_0–60 plasma_).

## Results

Brain activity uptake was low after injection of [^11^C]tariquidar with a mean AUC_0–60 brain_/AUC_0–60 plasma_ of 0.14 ± 0.03. At 60 min after radiotracer injection, 78 ± 12% of total radioactivity in plasma was in the form of unchanged parent radiotracer. Less than 1% of the total injected dose excreted in urine over 90 min.

## Conclusions

Low brain uptake of radioactivity is consistent with tariquidar being, at microdoses, a dual substrate of Pgp and BCRP. [^11^C]Tariquidar PET after inhibition of Pgp with unlabelled tariquidar may be a promising approach to selectively assess BCRP function at the human blood-brain barrier.

